# Demographic and Clinical Predictors of Trait Impulsivity in Parkinson's Disease Patients

**DOI:** 10.1155/2018/9472120

**Published:** 2018-04-19

**Authors:** Maddeson Riley, Megan Bakeberg, Michelle Byrnes, Alexa Jefferson, Soumya Ghosh, Rick Stell, Frank L. Mastaglia, Dana Hince, Ryan S. Anderton

**Affiliations:** ^1^School of Health Sciences, University of Notre Dame Australia, Fremantle, WA, Australia; ^2^Perron Institute for Neurological and Translational Science, Nedlands, WA, Australia; ^3^Centre for Neuromuscular and Neurological Disorders, University of Western Australia, Nedlands, WA, Australia; ^4^Institute of Immunology and Infectious Diseases, Murdoch University, Perth, WA, Australia; ^5^Institute for Health Research, University of Notre Dame Australia, Fremantle, WA, Australia

## Abstract

**Background:**

Impulsive behaviour has become increasingly recognised as a neuropsychiatric complication of Parkinson's disease (PD). Thought to be a product of compromised cognitive control, the spectrum of impulsive behaviours in PD ranges from cognitive disinhibition to impulse control disorders (ICDs).

**Objective:**

At present, there are no indicators for trait impulsivity in PD. The objective of the current study was to identify demographic and clinical predictors of susceptibility to trait impulsivity in a cohort of PD patients.

**Methods:**

The current study assessed impulsivity using the Barratt Impulsiveness Scale 11 (BIS-11) in a cohort of 87 PD patients. General linear models (GLMs) were used to identify clinical and demographic variables predictive of heightened BIS-11 second-order attentional and nonplanning subscale scores.

**Results:**

Male gender, no history of smoking, postsecondary education, and heightened disease severity were predictive of increased BIS-11 attentional scores (*p* < 0.05). Similarly, male gender, after secondary education, and disease severity were predictive of increased BIS-11 nonplanning scores (*p* < 0.05). Contrary to previous reports, dopaminergic medication use was not a significant determinant of either BIS-11 subscale scores.

**Conclusions:**

Several demographic and clinical variables including male gender, no history of past smoking, after secondary education, and elevated disease severity are associated with impulsivity in PD.

## 1. Introduction

In recent times, a variety of impulsive behaviours, often ranging in severity, have been reported as psychiatric complications associated with Parkinson's disease (PD) [1]. As a construct, impulsivity is broadly defined as the tendency to act prematurely or without foresight, with little regard for the often-negative consequences associated with these actions [2].

Previous studies suggest that PD patients display heightened impulsivity, particularly when taking dopaminergic medication [1, 3]. In addition to this, PD may impair response inhibition, an integral element of impulsivity, with PD patients performing worse on a range of measures of inhibition, including the Stop-Signal Task [4, 5], Go/No-Go [6], and antisaccade [7].

Impulsivity is thought to underlie several psychiatric disorders described in PD, particularly impulse control disorders (ICDs) including pathological gambling, compulsive shopping and eating, and hypersexual behaviours [1, 8, 9]. These behaviours represent extreme manifestations of impulsivity thought to be provoked by dopamine agonist [10] therapy and identified in only 13% of PD patients [11]. Despite the strong association between impulsivity and ICDs, PD patients may display impulsivity even in the absence of an ICD [8, 12]. Additionally, impulsivity represents a significant risk factor for the onset of an ICD, which further suggests that impulsivity in PD can be represented on a spectrum; beginning with mild cognitive and motor disinhibition and eventually manifesting as clinically identifiable ICDs [9].

Previous studies have identified several variables associated with ICDs including male gender [11], cigarette smoking history [11, 13, 14], dopamine agonist use [11, 13], younger age of disease onset [14, 15], a history of drug or alcohol abuse [9, 15], and a novelty-seeking personality [9, 15]. However, there are no current demographic or clinical indicators of future impulsivity in PD. Given the strong association between impulsivity and ICDs in addition with the absence of treatment options for impulsive behaviour, the identification of risk factors for trait impulsivity serves as a valuable clinical tool in optimising the prevention of impulsivity and, in turn, the future onset of ICDs.

As such, the present study characterised a heterogeneous cohort of Western Australian PD patients with the objective of identifying demographic and clinical predictors for the development of trait impulsivity. Trait impulsivity was assessed using the Barratt Impulsiveness Scale 11 (BIS-11), which has been used extensively in PD clinical settings. The BIS-11 is a reliable measure of trait impulsivity, addressing the complexity of impulsivity as a construct with a three-component conceptualization comprising attentional impulsivity (reduced ability to focus on the task at hand and racing thoughts), motor impulsivity (the tendency to act without thinking), and nonplanning impulsivity (lack of future- or fore-thought) [16]. Previous studies have shown adequate internal consistency of BIS-11 second-order attentional and nonplanning subscales with Cronbach's *α* of 0.74 and 0.72, respectively [17]. However, internal consistency of the BIS-11 second-order Motor subscale is below adequate with a Cronbach's *α* of 0.59 [17]. Further, the aim of the current study was to assess cognitive domains of impulsivity in PD, rather than motor domains. Therefore, in the current study assessed only subjects BIS-11 attentional and nonplanning scores.

## 2. Methods

### 2.1. Subjects

Eighty-seven home-based PD patients (54 males) were sequentially recruited from the Movement Disorders Clinic at the Perron Institute for Neurological and Translational Science (Perth, Australia) between 2008 and 2015. All patients were examined by a movement disorder neurologist prior to inclusion in the study for verification of the diagnosis in accordance with the UK Brain Bank criteria for IPD. All patients were taking levodopa, and 45 patients were on a dopamine agonist (pramipexole, rotigotine, and apomorphine). For medication dosages, LEDD was calculated from patient medication dosages as per the following: LEDD = (regular levodopa dosage) + 0.75 (slow-release levodopa) + 10 (bromocriptine) + 10 (pergolide) + amantadine. Fourteen patients had undergone deep brain stimulation (DBS) therapy. Demographic variables including a history of smoking and patients' level of education were also collected.

### 2.2. Clinical Assessments and Impulsivity Screening

All clinical assessments were evaluated in the “ON” state. Motor symptoms were evaluated using the MDS-Unified Parkinson's Disease Rating Scale (MDS-UPDRS) Part III and Hoehn and Yahr Scale. In addition, each participant was evaluated by a clinical psychologist and completed a battery of neuropsychological assessments, as previously described [18]. The Mini-Mental State Examination (MMSE) was used to determine patients' cognitive status, with an MMSE score of less than 26 indicating “cognitive impairment” and greater than 26 indicating “normal” cognition. The “Barratt Impulsivity Scale 11” (BIS-11) was employed as a validated self-report questionnaire for screening impulsivity. The BIS-11 consists of 30 questions scored on a four-point scale, with each item corresponding to one of the three BIS-11 second-order subscales. The current study aimed to investigate cognitive domains of trait impulsivity rather than motor impulsivity and therefore only BIS-11 second-order attentional and nonplanning subscale scores are presented. Questions corresponding to these subscales are summarised in Table 1. Overall BIS-11 scores were calculated as the sum of these 30 scores (to yield a score out of 120), with higher scores indicating greater impulsivity. The sum of second-order BIS-11 attentional and nonplanning items was used to calculate patients' BIS-11 second-order subscale scores. BIS-11 second-order attentional and nonplanning scores were scored out of 32 and 44, respectively. During the screening process, patients displaying any signs of an impulse control disorder were invited to seek further consultation with the clinical psychologist.

### 2.3. Statistical Methods

Data were analysed using IBM-SPSS (v. 24, IBM Corporation). A significant nominal *p* value of <0.05 was employed. Univariate regression analysis of continuous demographic and clinical variables (MDS-UPDRS III, disease duration, and LEDD) and categorical demographic variables and clinical variables (gender, smoking history, level of education, i.e., secondary school or tertiary education, and cognitive status) was performed in order to determine whether these variables were significantly associated with BIS-11 second-order attentional and nonplanning subscale scores. Level of education was dichotomised into secondary school, referring to participants who reported not progressing past secondary school, and tertiary education, referring to participants who reported entering into a tertiary level of education. Participants who reported entering into a tertiary level of education were considered as having a higher level of education.

General linear models (GLMs) were used to analyse the relationship between variables identified as being significant in the univariate models and the two BIS-II second-order subscales. Variables proposed to be risk factors for the development of impulse control disorders in PD were also included in the GLMs, despite not displaying statistical significance in the univariate models. Variables included in the GLMs were gender, smoking history, dopamine agonist usage, LEDD, DBS history, age at disease onset, disease duration, MDS-UPDRS III scores, cognitive status, and participants' level of education. Nonsignificant variables were removed singularly in order of least significance until the final models were determined.

## 3. Results

### 3.1. Cohort Information and Clinical Data

Mean demographic details and the results of clinical assessments are shown in Table 2. The predominantly male cohort ranged in age and disease duration. At the time of the study, a total of 45 participants were medicated with dopamine agonists (DAs), and 14 participants had undergone DBS therapy. When examined according to MMSE score, 70 patients did not display cognitive impairment, and 17 were classified as being cognitively impaired. Overall, the cohort displayed a mean total BIS-11 score of 62.5 (±8.9) and had no prior history of impulsivity, based on information provided during consultation with a clinical psychologist.

### 3.2. Univariate Association of Demographic and Clinical Variables with Second-Order Attentional and Nonplanning Impulsivity

Univariate regression models revealed selected clinical and demographic variables associated with BIS-11 second-order attentional scores including gender (*p*=0.048) and MDS-UPDRS III rating (*p*=0.005). The residuals from these univariate models were normally distributed. The patients' level of education was positively associated with BIS-11 second-order attentional scores; however, this association did not reach statistical significance (*p*=0.076). BIS-11 second-order nonplanning scores were significantly associated with gender (*p*=0.001), MDS-UPDRS III rating (*p*=0.001), and disease duration (*p*=0.003). While the residual values of the univariate models for MDS-UPDRS III rating and disease duration were normally distributed, those for gender were not.

### 3.3. Demographic and Clinical Predictors of Second-Order Attentional Impulsivity in Multivariate Models

**Figure 1:**
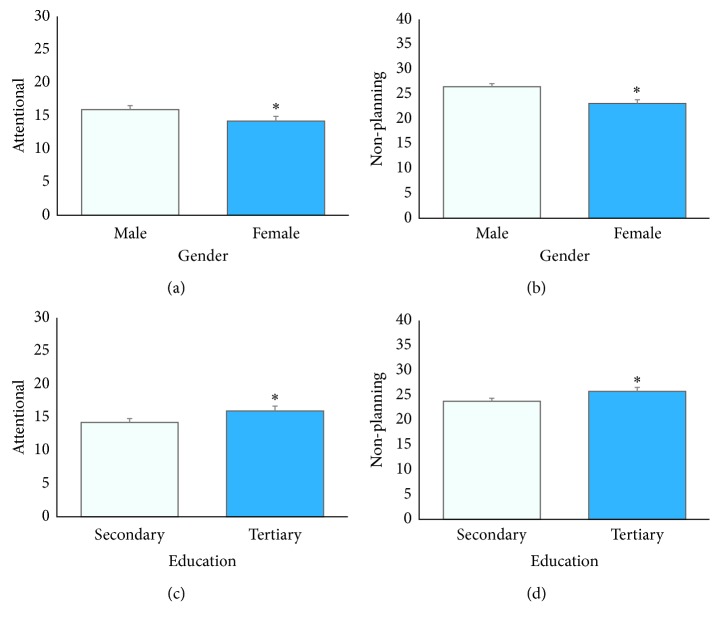
Estimated marginal mean (EMM) BIS-11 second-order subscale scores. EMM BIS-11 (a) attentional and (b) nonplanning scores were significantly higher in male subjects. EMM BIS-11 (c) attentional and (d) nonplanning scores were significantly higher in those who had pursued tertiary education when compared with those who had attained a secondary level of education.

Multivariate general linear models were fitted to identify demographic and clinical determinants of BIS-11 second-order attentional and nonplanning subscale scores. The residual values for these multivariate models were normally distributed. Dopamine agonist usage, LEDD, DBS surgery, age at onset, disease duration, and cognitive status (cognitively impaired and not cognitively impaired) were found not to be significant predictors of BIS-11 second-order attentional or nonplanning subscale scores. In contrast, being of male gender, no history of smoking, higher level of education, and increased disease severity are predictive of elevated BIS-11 second-order attentional subscale score when entered simultaneously in the model (Table 3). Specifically, male participants were predicted to score 1.8 points higher than female participants (*p*=0.011). The estimated marginal mean (EMM) BIS-11 attentional score for male participants was 15.95 and 14.22 for female participants (*p*=0.019) (Figure 1). Patients with no smoking history were predicted to score 2.2 points higher than those with a history of smoking (*p*=0.038), and patients who had pursued tertiary studies were predicted to score 1.7 points higher than those who had attained a secondary level of education (*p*=0.023) on the BIS-11 second-order attentional subscale. The EMM BIS-11 attentional score for those who undertook a secondary level of education was 14.22 and 15.95 for those who pursued tertiary studies (*p*=0.023) (Figure 1). In addition to this, for every additional MDS-UPDRS III point, BIS-11 second-order attentional scores were predicted to rise by 0.07 points (*p*=0.003). For example, a male patient who did not report a history of smoking was predicted to score 2.2 points higher than a female patient who did report a history of smoking.

### 3.4. Demographic and Clinical Predictors of Second-Order Nonplanning Impulsivity in Multivariate Models

Similarly, multivariate general linear models indicated that being of male gender, greater level of education, and increased disease severity were predictive of elevated BIS-11 second-order nonplanning subscale scores (Table 4). Smoking history was not found to be a significant determinant of nonplanning subscale scores. Specifically, male participants were predicted to score three points higher than female participants (*p*=0.002). The EMM for BIS-11 nonplanning scores was 26.45 for male participants and 23.09 for female participants (*p*=0.001). Those who had pursued tertiary education scored two points more than those who had attained a secondary level of education (*p*=0.047) on the BIS-11 second-order nonplanning subscale. The EMM for BIS-11 nonplanning scores was 23.79 for those who had attained a secondary level of education and 25.76 for those who had pursed tertiary education (*p*=0.047) (Figure 1). In addition to this, participants were predicted to score 0.1 points higher for every additional MDS-UPDRS III score (*p*=0.001) on the BIS-11 second-order nonplanning subscale. For example, a patient who obtained an MDS-UPDRS III rating of 50 was predicted to score 3.3 points higher on the BIS-11 second-order nonplanning domain than a patient who obtained an MDS-UPDRS III rating of 20.

## 4. Discussion

Several risk factors for the onset of ICDs in PD subjects have been identified, including gender [11], smoking history [11, 13, 14], history of drug or alcohol abuse [9, 15], impulsivity [9], and a novelty seeking personality [9, 15]. However, there are no established demographic or clinical indicators for trait impulsivity in PD subjects. In light of the close relationship between trait impulsivity and the development of ICDs, and in conjunction with the absence of treatments for impulsive behaviours, there is a growing need to identify risk factors associated with impulsivity. Our findings suggest that several demographic and clinical variables may underlie impulsive behaviours in PD subjects.

Aligning with previous findings regarding PD-ICD subjects, the current study suggests that being of male gender is a significant risk factor for the development of trait impulsivity [19, 20]. Male subjects displayed significant associations between disease severity and the BIS-11 second-order attentional and nonplanning subscales. Regression analysis revealed that male subjects were predicted to score higher, particularly in the BIS-11 second-order nonplanning subscale, when compared with female subjects. A similar gender-related pattern of impulsive behaviour has been demonstrated in both PD-ICD subjects and the general population. For instance, Kenangil et al. [20] reported that among a cohort of 554 PD patients, 33 had a diagnosed ICD, of which 81% were men. In addition to this, male PD subjects are more likely to develop pathological gambling and hypersexual behaviours than females, with a similar pattern also evident in the general population [11].

This predisposition to developing impulsive behaviours in males may be related to men historically requiring greater stimulation and therefore being more likely to engage in sensation-seeking and impulsive behaviours than females [21]. Studies exploring gender differences in impulsivity have underlined that whilst females display heightened punishment sensitivity and are therefore more likely to avoid danger or risk, males do not exhibit this trait which may underlie the male propensity to partake in risky behaviours and therefore display heightened impulsivity [22]. It is important to note, however, that male PD subjects are less likely to develop compulsive eating and shopping disorders than female PD subjects, thus indicating that the type of impulsive behaviour reported may be gender-specific [11].

To our knowledge, this is the first study to explore the influence of level of education on the development of impulsivity in PD. Given the association between lower education status and accelerated cognitive decline in PD, the current study investigated whether achieving lower level of education (i.e., finishing secondary school and not pursuing tertiary studies) may also be related to elevated trait impulsivity [23]. However, the present study found that pursuing tertiary education and therefore pursuing education after secondary school was a risk factor for elevated BIS-11 second-order attentional and nonplanning scores. Weintraub et al. [11] identified a similar trend in a cohort of 3,090 PD patients, with those diagnosed with an ICD more likely to have more formal education. We suggest that seeking and obtaining further education may reflect distortions in patients' reward processing, an important element of impulsivity, thereby explaining this association between higher education status and trait impulsivity. Neuroimaging studies have demonstrated that PD patients, particularly those taking dopaminergic medication or with a clinically diagnosed ICD, may exhibit blunted reward anticipation and experiencing [24, 25]. In turn, these patients often make riskier choices in order to compensate for this diminished reward response [24, 25]. Although seeking out additional education is not necessarily an inherently risky process, it may nonetheless be related to patients' reduced ability to process and experience reward, possibly leading to dissatisfaction with their current education status and in turn, stimulating patients to pursue further education opportunities with diminished disinhibition.

Heightened disease severity was also a significant determinant of elevated BIS-11 second-order attentional and nonplanning scores in the described cohort, suggesting a relationship between more severe motor symptoms and future trait impulsivity in PD. Indeed, previous studies have discounted disease severity as a risk factor for the development of ICDs in PD subjects. Instead, these studies have underlined an association between motor complications as measured by the MDS-UPDRS IV and the future onset of ICDs, associating this link with elevated dopaminergic loads as a result of dopaminergic medication usage [26, 27]. Despite this, a more recent investigation of impulsivity as a multidimensional rather than unitary construct (i.e., ICD±) has reported a link between elevated MDS-UPDRS III scores and impulsivity in a cohort of 30 PD patients [6]. In this study, linear regression analysis verified that increased disease severity was significantly associated with impaired performance on a variety of measures of impulsivity including the Frontal Assessment Battery, Stroop test, BIS-11, Stop-Signal Task (SST), Go/No-Go task, and the Cambridge Gambling Task [6]. We propose that the pathophysiological basis underlying this association may be related to deteriorating motor symptoms being reflective of progressive brain atrophy and dopaminergic depletion, which, in turn, has been shown to have implications for performance on a variety of measures of impulsivity and therefore may contribute to the development of impulsive behaviours [6].

Contrary to previous reports, an absence of past smoking was a significant risk factor for elevated BIS-11 second-order nonplanning scores. Although a history of smoking has been associated with the development of ICDs in PD subjects [11, 13], our findings suggest otherwise with regard to impulsivity, indicating an inverse relationship between past smoking and trait impulsivity. However, given only 14 participants reported a history of smoking, this finding is more likely attributed to the small sample size of smokers included in the current cohort, as opposed to the presence of a potential association between smoking history and impulsivity.

A growing body of evidence exists to support the notion that dopamine agonist [10] medication, administered to alleviate the motor symptoms of PD in fact provokes ICDs in PD subjects. DA usage has been associated with a 3.5-fold increased risk of developing an ICD in PD patients [11]. Although the mechanism underlying this association remains unclear, the ability of DAs to stimulate dopamine receptors in the striatal limbic system, an area involved in reward and motivation, may contribute to the development of reward-driven and compulsive behaviours exhibited by PD patients with ICDs [28]. Interestingly, dopaminergic medication was not a significant determinant of trait impulsivity in this PD cohort. Though dopaminergic medication use has been associated with ICDs in PD cohorts, the prevalence of ICDs in only a fraction of PD patients treated with DAs highlights the importance of underlying susceptibility unrelated to dopaminergic medication use in the development of impulsivity [11]. Further, a significant proportion of PD patients have been diagnosed with an ICD before the initiation of any antiparkinsonian treatment, supporting the notion of underlying individual susceptibility to ICDs [8]. Therefore, the current finding further underlines the importance of additional clinical and demographic factors in the onset and development of impulsivity in PD.

## 5. Limitations

A number of limitations of the current study must be acknowledged. Firstly, the self-report nature of the BIS-11 may introduce a degree of bias in the gathered responses due to patients often being less inclined to report impulsive tendencies. Although the BIS-11 is frequently used as a measure of impulsivity in PD studies, the BIS-11 was not originally designed for use in a PD clinical setting, thereby somewhat limiting the reliability of the BIS-11 second-order scales. As the presence of depression or anxiety was not noted and therefore patients with depression or anxiety were not actively excluded from the current study, the confounding effect of these psychiatric disorders on impulsivity outcomes was not controlled for. In addition to this, patients' cognitive status was determined using an MMSE cutoff score of 26, which may not have accurately identified all patients exhibiting signs of cognitive impairment. Further, available data relating to the level of patient education indicated the commencement of education after secondary school, but did not necessarily provide information on study completion. As such, this study cannot attribute tertiary education attainment to a rise in BIS-11 domain scores, rather the mere pursuit of tertiary education.

## 6. Conclusion

In conclusion, the current study identified several demographic and clinical indicators of increased trait impulsivity in PD including male gender, heightened disease severity, an absence of past smoking, and higher level of education. The identification of demographic and clinical indicators of trait impulsivity in PD patients may serve as a valuable clinical tool in facilitating the early recognition of impulsivity and determining patients' therapeutic regimen, particularly regarding whether dopaminergic therapy may provoke impulsive behaviours and likely ICDs in the future.

## Figures and Tables

**Table 1:** Questions corresponding to BIS-11 second-order attentional and nonplanning domains.

BIS-11 2nd order subscale	Corresponding questions
Attentional impulsivity	5. “I don't pay attention”
	6. “I have racing thoughts”'
	9. “I concentrate easily”
	11. “I squirm at plays or lectures”
	20. “I am a steady thinker”
	24. “I change hobbies”
	26. “I often have extraneous thoughts when thinking”
	28. “I am restless at the theatre or lectures”
Nonplanning impulsivity	1. “I plan tasks carefully”
	7. “I plan trips well ahead of time”
	8. “I am self controlled”
	10. “I save regularly”
	12. “I am a careful thinker”
	13. “I plan for job security”
	14. “I say things without thinking”
	15. “I like to think about complex problems”
	18. “I easily get bored when solving thought problems”
	27. “I am more interested in the present than the future”
	29. “I like puzzles”

**Table 2:** Baseline clinical characteristics of the PD cohort (*n*=87) used in this study.

Clinical characteristic	Mean (SD) or *n* (%)
Gender	
Male	54 (62%)
Female	33 (38%)
Age (years)	62.8 (9.2)
Age of onset (years)	53.3 (10.2)
Disease duration (years)	10.4 (6.8)
Dopamine agonist usage	
Yes	45 (51.7%)
No	42 (48.3%)
LEDD (mg)	671 (389)
Deep brain stimulation	
Yes	14 (16.1%)
No	73 (83.9%)
Smoking history	
Yes	13 (14.9%)
No	74 (85.1%)
Level of education	
Secondary school	58 (66.7%)
Tertiary education	29 (33.3%)
MDS-UPDRS III (motor)	18 (14.2)
Cognitive status	
Cognitively impaired	22 (25.3%)
Not cognitively impaired	65 (74.7%)
BIS-11 scores	
Attentional 2nd order	15.8 (3.7)
Nonplanning 2nd order	24.8 (4.9)
Total score	62.5 (8.9)

**Table 3:** Final multivariate model parameter estimates: predictors of BIS-11 second-order attention subscale scores.

Variable	*β* coefficient	Std. error	*p*
(Intercept)	12.537	1.3095	0.000
Gender			
Male	1.730	0.7401	0.019
Female	0^∗^	—	—
Smoking history			
Yes	−2.207	1.0318	0.032
No	0^∗^	—	—
Level of education			
Secondary school	0^∗^	—	—
Tertiary education	1.730	0.7581	0.023
MDS-UPDRS III	0.080	0.0253	0.002

^∗^Comparison category set to zero.

**Table 4:** Final multivariate model parameter estimates: predictors of BIS-11 second-order nonplanning subscale scores.

Variable	*β* coefficient	Std. error	*p*
(Intercept)	22.154	1.0648	0.000
Gender			
Male	3.366	0.9680	0.001
Female	0^∗^	—	—
Level of education			
Secondary school	0^∗^	—	—
Tertiary education	1.971	0.9936	0.047
MDS-UPDRS III	0.106	0.0328	0.001

^∗^Comparison category set to zero.
